# Improving Negative Emotion Recognition in Young Offenders Reduces Subsequent Crime

**DOI:** 10.1371/journal.pone.0132035

**Published:** 2015-06-29

**Authors:** Kelly Hubble, Katharine L. Bowen, Simon C. Moore, Stephanie H. M. van Goozen

**Affiliations:** 1 School of Psychology, Cardiff University, Cardiff, United Kingdom; 2 Violence Research Group, School of Dentistry, Cardiff University, Cardiff, United Kingdom; 3 Department of Clinical Child and Adolescent Studies, Leiden University, Leiden, Netherlands; University of Lincoln, UNITED KINGDOM

## Abstract

**Background:**

Children with antisocial behaviour show deficits in the perception of emotional expressions in others that may contribute to the development and persistence of antisocial and aggressive behaviour. Current treatments for antisocial youngsters are limited in effectiveness. It has been argued that more attention should be devoted to interventions that target neuropsychological correlates of antisocial behaviour. This study examined the effect of emotion recognition training on criminal behaviour.

**Methods:**

Emotion recognition and crime levels were studied in 50 juvenile offenders. Whilst all young offenders received their statutory interventions as the study was conducted, a subgroup of twenty-four offenders also took part in a facial affect training aimed at improving emotion recognition. Offenders in the training and control groups were matched for age, SES, IQ and lifetime crime level. All offenders were tested twice for emotion recognition performance, and recent crime data were collected after the testing had been completed.

**Results:**

Before the training there were no differences between the groups in emotion recognition, with both groups displaying poor fear, sadness and anger recognition. After the training fear, sadness and anger recognition improved significantly in juvenile offenders in the training group. Although crime rates dropped in all offenders in the 6 months following emotion testing, only the group of offenders who had received the emotion training showed a significant reduction in the severity of the crimes they committed.

**Conclusions:**

The study indicates that emotion recognition can be relatively easily improved in youths who engage in serious antisocial and criminal behavior. The results suggest that improved emotion recognition has the potential to reduce the severity of reoffending.

## Introduction

Antisocial behaviour in childhood and adolescence is associated with a range of negative outcomes in adulthood. It predicts future antisocial behaviour (arrests, severity of crimes, conviction rates and length of prison sentences [1]), substance abuse and dependence in adulthood, early pregnancy in girls, persistent health problems, and psychiatric illness [2, 3]. These negative outcomes are costly to society, as well as to the individuals themselves [4]; there are high costs not only because of the crimes committed, the extra educational provision required, the foster/residential care needed, and other state benefits during adolescence, but also because of the associated mental and physical health problems of antisocial behaviour in adulthood [5]. For these reasons intervention strategies and support for young people with antisocial behavioural problems are highly desirable.

The short- and long-term effectiveness of individual-focused preventions (e.g., child skills training) and family-focused preventions (e.g., Multi-Systemic Therapy) in reducing antisocial behaviours has been demonstrated [6]. Most of the evidence for the effectiveness of interventions comes from the USA; evidence from the UK has been criticised for being less robust in terms of both the quantity and quality of interventions tested [7]. Given that statutory Youth Offending Services (YOS) in the UK are arguably more supportive than those in the USA [8], it is important that the effectiveness of interventions is tested within the context of current provisions. Similarly, whilst some interventions are successful, reoffending data also show that they do not work for everyone. For example, family-focused interventions are highly intensive and require involvement of family members, which is often problematic in young offenders [9]. In addition, some young offenders may experience specific problems that are not targeted by standard interventions. The high persistence and poor prognosis associated with childhood antisocial behaviour, coupled with the limited effectiveness of current treatments, are the main reasons why the neuropsychological and neurobiological correlates of antisocial behaviour in childhood should be given more attention in terms of designing targeted interventions [10].

One of the best-replicated findings is that individuals who exhibit inappropriate interpersonal and antisocial behaviour have problems in facial emotion recognition, particularly fear and sadness [11]. According to Blair’s Integrated Emotion System (IES) model [12], amygdala dysfunctions can impair the ability to correctly process others’ distress related cues and thereby contribute to antisocial behaviour. Thus, if a person cannot correctly identify the distress they are causing to another person, they are more likely to continue with the behaviour that is causing the harm. Consistent with this theory empirical studies have found specific fear and sadness recognition impairments among clinical and community samples of antisocial individuals, including psychopathic adults [13, 14], children high in psychopathic traits [15], adolescents with conduct disorder [16], adolescents with mental health problems [17], and antisocial adolescents recruited from mainstream schools [18] or the community [19]. Nevertheless, a recent meta-analysis indicates that a more general facial emotion recognition impairment is evident in psychopathy [20]. Indeed, problems with disgust [21] and anger have also been observed [22], with impairments in anger recognition in particular being reported in adolescents who display antisocial behaviour [17], adolescents with early-onset conduct disorder [16], and juvenile offenders [19]. Evidence of pervasive deficits, combined with evidence suggesting boys with conduct problems show impairments in allocating attention to emotionally salient stimuli [23], has led to the proposition that a more general dysfunction in attentional mechanisms underlies the facial emotion recognition deficits in those who show antisocial behaviour [18].

Finally, it has been shown that delinquents are more likely than non-delinquents to misinterpret expression of disgust as anger [24], and ambiguous or neutral expressions as negative [25]. A bias towards anger (i.e., a hostility bias), as opposed to a deficit in anger recognition, may lead one to expect more dangerous and threatening situations, and thus contribute to antisocial behaviour. A recent study in young offenders suggests that both accounts may be true. Bowen et al. [19] found evidence to suggest than young offenders were better than controls in detecting high intensity angry faces, but were impaired in detecting low intensity ones. Since angry faces serve as warning signals of social punishment, young offenders may be less sensitive to low intensity or early warning signals and thus continue to behave in socially unacceptable ways. Targeted emotion recognition interventions [26] could reduce these biases and also improve the ability to detect more subtle emotional expressions. This approach might contribute towards reducing antisocial behaviour.

Training programs that teach participants to pay more attention to important facial features using explicit methods [27, 28]–which specifically remind participants to look at key facial areas—or implicit methods [29]–which do not explicitly mention key facial areas, but train participants to examine these areas by following a dot probe—demonstrate that facial emotion recognition can be modified. Two recent studies indicate that emotion training could also be effective in young people with behavioural problems. Dadds, Cauchi, Wimalaweera, Hawes and Brennan [30] found no beneficial effect of training on parent and teacher reports in an offender group as a whole, but the behaviour of those with callous-unemotional (CU) traits was judged to have improved. Penton-Voak et al. [31] succeeded in modifying emotional cognitive biases of angry ambiguous expressions in aggressive youths, who subsequently reported fewer aggressive behaviours in the two weeks after intervention.

If young offenders’ facial emotion recognition of key distress emotions like fear and sadness can be improved through training, then according to the IES model such interventions could alter how young offenders respond and interact with potential victims. Similarly, by improving the detection of anger, young offenders may pick up early warning signals of social punishment more effectively and refrain from continuing their negative actions. On the other hand, an improved ability to detect anger may reduce misinterpretation of other emotions, such as disgust, and lead to less threatening responses in otherwise non-threatening situations. Consequently, the training of these emotions could have positive effects on future antisocial behaviour, ultimately leading to a reduction in crime.

The current study assessed facial affect recognition and objectively recorded crime levels in a group of juvenile male offenders prior to and after completion of an emotion recognition training intervention and compared their data to those of juvenile male offenders who did not complete the training intervention. We expected (a) that the training would result in a significant improvement in the recognition of those negative emotions that were trained (fear, sadness, anger), and (b) that offenders in the training condition would show a greater reduction in reoffending levels compared to those receiving the usual treatment up to six months post training.

## Method

### Participants

Male young offenders (YOs) aged 12–18 years (mean = 16.21) took part and were recruited from the Cardiff and Vale of Glamorgan Youth Offending Services (YOS). Young offenders were recruited with the help of YOS caseworkers who recommended suitable participants. All participants completed the emotion recognition test twice (average time between tests = 23 days). YOs were eligible to participate if they had been convicted of an offence and received a court order to attend the YOS. To be included in the analyses participants were required to have completed the pre- and post- facial recognition measures in the required time frame without incarceration; however, later incarceration was not a reason for exclusion. All participants met these eligibility requirements and no participants dropped out from the study. A group of 24 offenders (Training group) completed the emotion training in the interval between the first and second emotion recognition test; another group of 26 offenders (Control group) was tested twice during the same time period, but did not receive the emotion training. Group allocation was strongly influenced by the opportunity and availability of the offenders to attend the YOS offices where testing took place for the required number of sessions. This was discussed with caseworkers before participants were officially approached about the study. Participants were specifically asked to take part in the Training group or the Control group, and were not given the option to choose between conditions. All parts of the study were completed at YOS offices and conducted by trained researchers (KH, KLB). Sample size (n = 50) was based on a previous study comparing YOs and non-offending matched controls [19].

### Ethics Statement

The study was approved by the School of Psychology Research Ethics Committee at Cardiff University. All participants (n = 50) and their parents/guardians provided written informed consent.

### Measures and Materials

#### Crime Data

Data on the number and severity of offences that YOs had committed and which had led to criminal prosecutions by a court were compiled from YOS databases after the completion of the emotion recognition/training study. For each offender we collected crime data covering three time periods: (1) lifetime crime data; these covered all crimes ever committed up to 12 months ago; (2) pretest crime data; all crimes committed in the 6 months leading up to the first emotion recognition test, and (3) posttest crime data; all crimes committed in the 6 months following the second emotion recognition test. Juvenile offenders had been involved in the following types of offence: aggravated burglary, aggravated taking, arson, assault, attempted robbery, attempted theft, breach of order, burglary, public order (harassment, affray), public order (common assault), criminal damage, motor/traffic offences, obstructing police, drug offences, robbery, shoplifting, theft, public order (threatening, abusive), trespassing, TWOC (taking [vehicle] without owner’s consent), wounding with intent to do GBH (grievous bodily harm; i.e., assault). The YOS also assigns each offence a severity score ranging from 1 (e.g., minor public order offences) to 8 (e.g., murder). In case of multiple offences, the highest severity score (the most serious crime committed) was recorded. The mean number of offences in our sample before the start of our study (lifetime offence rate) was 6.68 (SD = 8.75; range 1–49), the mean severity score was 3.20 (SD = 1.09; range 1–6), and the mean score of the most severe offence committed was 4.82 (SD = 1.66; range 1–7).

#### SES and IQ

Many of the offenders were not in statutory full time education, but received some form of education provided by the YOS. Because of this it was considered to be more appropriate to use measures of IQ to assess educational ability. The two-subset form of the Wechsler Abbreviated Scale of Intelligence (WASI; [32]) was used to provide an estimated IQ score. Twenty offenders in the training group and 17 in the control group provided IQ data. Socio-economic status (SES) was estimated using the National Statistics estimates of average household total weekly income based on each participant’s postcode (Low = £0–£520; Middle = £521–£670; High = £671+).

#### Personality Measures

The Youth Psychopathy Inventory (YPI [33]) is a 50-item validated youth self-report questionnaire that assesses general psychopathic tendencies. The YPI is scored on a 1–4 Likert scale, giving a sum score between 50–200. A higher score is indicative of higher levels of psychopathic traits. The YPI is made up of four subscales. Given the relevance of CU traits in antisocial behaviour we also examined the Callous and Unemotional subscale separately. A total score was calculated, which could range between 15–60 with a higher score being indicative of higher CU traits. Forty-two young offenders completed this measure (Training = 22; Control = 20); 8 participants were unable or unwilling to complete the questionnaire.

#### Facial Emotion Recognition Measure

The Facial Emotion Recognition (FER; see [19] for details) measure consists of a series of 150 slides presented on a laptop, displaying facial expressions. Six target faces—three male and three female—were used. Each target displays a neutral expression or one of five basic emotions (happy, sad, anger, fear or disgust); emotional expressions were morphed with their corresponding neutral expression (0% emotion) to display faces at 25%, 50%, 75% and 100% emotional intensity. The question “What emotion is this person showing?” accompanies the target image, along with numbered and labeled options. Percentage correct recognition scores for each emotion were calculated.

#### YOS Intervention

Participants were known to the YOS, had their own caseworker and continued to receive their statutory interventions and allocated contact time with the YOS for the duration of the study (including the 6 months follow-up). The type of intervention and amount of contact with the caseworker varied between offenders depending on the court order received and their risk of reoffending.

Ten YOs (training = 4; control = 6) belonged to preventative or early intervention programmes. Offenders on these orders are considered at low level of risk of reoffending and are seen less regularly. Thirty-four participants had a Youth Rehabilitation Order (training = 16; control = 18), which is the standard community sentence for the majority of offenders. The specific requirements of the order and the amount of contact with the YOS follow an individual risk- and needs-based approach. Six participants were on probation after a custodial sentence or on bail (training = 4, control = 2). Offenders on these orders are considered at high risk and are seen 3–5 times a week. Although all interventions are tailored to each individual, most orders encourage engagement with statutory education and will work with the young person to put this in place if it is not currently happening (for example, through training, work placements, apprenticeships and college), as well as with health, substance misuse and family support services, and focus on restorative justice and encourage victim work.

#### Emotion Training

We used an adapted version of the Facial Affect Recognition (FAR) intervention [28], a protocol-based computerised intervention designed to train participants to identify the emotional expressions of happiness, sadness, fear and anger. The FAR consists of several levels of emotion tasks. For example, tasks require participants to identify the emotional expression of a face, to describe an event that has made them feel that emotion, and mimic the emotion using a mirror. Tasks also require participants to focus on specified features of an emotional face and select the correct description of that feature from several options. Difficulty in correctly identifying the emotional expression gradually increases throughout the intervention by using lower intensity facial expressions and by using fewer cues to guide participants’ attention. All elements of the FAR were completed, however the delivery time was adapted to take into consideration that YOs generally completed the intervention much faster than participants in previous studies [28] and could only been seen once a week. Participants completed the tasks within two to three sessions over a 2-week period. Total training time was approximately 2 hours.

### Statistical methods

The FER variables are mean correct recognition scores for happy, sad, fearful, disgust and angry expressions, at both pre- and post-test. A mixed-design ANOVA was used with emotion (5 levels), and time (2 levels) as within-subject factors, and group (Training vs. Control) as between-subject factors. Where the assumption of sphericity was violated the Huynh-Feldt correction was used. Planned follow-up tests explored the effect of training on each emotion separately and used the Bonferonni correction. One-way ANOVAs examined differences in age, IQ, SES and personality measures between the experimental groups. Time to reoffend was analysed using the Cox Proportional Hazards Model [34]. To compare mean offence rates of the training and control group and their severity scores 6 months after the training, general linear models were used. To adjust for the possibility of regression to the mean, baseline scores were subtracted from mean posttest scores and included as a covariate [35]. To explore count data such as the total number of offences committed, negative binomial regression was used. The dataset underlying the reported findings is available in S1 Dataset.

## Results

Chi-square analysis revealed no differences between the Control and Training groups with respect to type of orders and interventions received, χ^2^(2) = 1.1, *p* = .58, and no differences in the amount of contact participants had with the YOS for the duration of the study, χ^2^(2) = 3.63, *p* = .16.

### Demographic characteristics and pre-training crime data

Table 1 shows the demographic characteristics and the offence data for both groups. Chi-squared analysis revealed the groups did not differ in socio-economic status, χ^2^[2] = 2.03, *p* = 0.36. One-way ANOVAs indicated that the Training and Control groups did not differ in age, *F*[1, 49] = 0.59, *p* = .45, IQ, *F*[1, 35] = 0.96, *p* = .33, psychopathic traits, *F*[1, 41] = 3.04, *p* = .09, CU traits, *F*[1, 41] = 0.33, *p* = .57, age of first offence, *F*[1, 48] = 3.58, *p* = .07, or lifetime offending rate, *F*[1, 49] = 0.04, *p* = .84. Although the groups did not differ in offending rate, *F*[1, 49] = 2.74, *p* = .10, or crime severity, *F*[1, 49] = 2.06, *p* = .16, in the 6 months leading up to the emotion recognition study, they differed in terms of their most severe offence, with YOs in the Training group committing marginally more serious offences, *F*[1, 49] = 3.88, *p* = .055; η^2^ = .08.

**Table 1:** Demographic characteristics and offending data of young offenders.

Variable	Training	Control
Age (years)	16.08 (1.2)	16.35 (1.2)
IQ	80.89 (9.64)	83.77 (7.64)
SES (mean)	1.3 (0.62)	1.6 (0.86)
Low (= 1)	79% (19)	65% (17)
Middle (= 2)	13% (3)	12% (3)
High (= 3)	8% (2)	23% (6)
YPI	115.2 (16.5)	125.8 (22.8)
CU traits	34.2 (6.6)	35.5 (8.4)
Age at first offence	14.17 (1.7)	13.24 (1.7)
Lifetime offence rate	6.42 (6.24)	6.92 (10.68)
Pretest offence rate (6m pretest)	3.63 (4.95)	1.92 (1.67)
Pretest offence most severe (6m pretest)	3.75 (2.23)	2.62 (1.84)
Pretest offence mean severity (6m pretest)	2.92 (1.82)	2.24 (1.53)
Re-offence rate (6m posttest)	1.67 (2.58)	0.92 (1.44)
Re-offence most severe (6m posttest)	2.08 (2.28)	1.85 (2.53)
Re-offence mean severity (6m posttest)	1.76 (1.89)	1.57 (2.11)

Table entries show mean values (standard deviations in parentheses), or % of group (with numbers in parentheses); IQ = intelligence quotient; SES = socio-economic status; YPI = Youth Psychopathy Inventory; CU = Callous and Unemotional.

### Emotion recognition: Training and Control group comparisons

**Fig 1:**
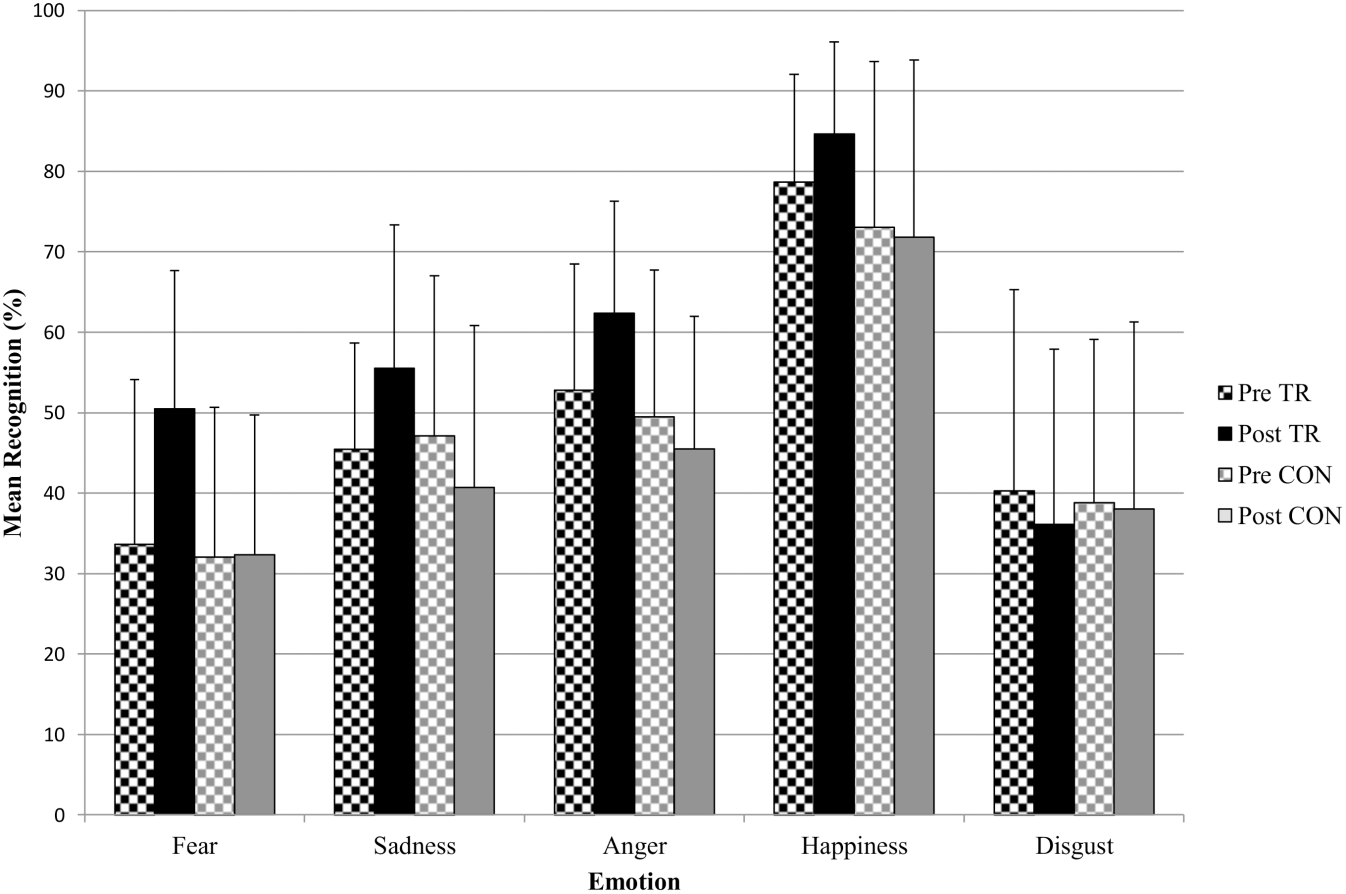
Mean fear, sadness, anger, happiness and disgust recognition scores for young offenders in the Training (TR) or Control (CON) group at pretest (Pre) and retest (Post). Error bars show +1 standard deviation.

Fig 1 shows pretest and retest scores on fear, anger, sadness, happiness and disgust for the Training and Control groups. The three-way interaction between group, emotion and time was significant, *F*[4,164] = 2.44, *p* = .05, η^**2**^ = .01. We therefore next examined the effect of time and condition on each emotion separately.

#### Fear

There was a significant time by group interaction, *F*[1, 48] = 13.00, *p* = .001, η^2^ = .17. Simple effects tests revealed fear scores did not differ between groups at pretest, *F*[1, 48] = 0.24, *p* = .63, η^2^ = .00; however, at posttest the Training group performed significantly better, *F*[1, 48] = 20.46, *p* < .001, η^2^ = .30. The Training group significantly improved from pre- to posttest, *F*[1, 48] = 25.91, *p* < .001, η^2^ = .35, whereas there was no difference as a function of time in the Control group, *F*[1, 48] = 0.09, *p* = .93, η^2^ = .00.

#### Sadness

There was a significant time by group interaction, *F*[1, 48] = 14.30, *p* < .001, η^2^ = .23. Simple effects tests revealed that sadness scores did not differ between the groups at pretest, *F*[1, 48] = 0.09, *p* = .77, η^2^ = .00, but the Training group performed significantly better at posttest, *F*[1, 48] = 8.89, *p* = .004, η^2^ = .16. The Training group significantly improved from pre- to posttest, *F*[1, 48] = 10.93, *p* = .002, η^2^ = .19, whereas the Control group performed worse over time; *F*[1, 48] = 4.07, *p* = .049, η^2^ = .08.

#### Anger

There was a significant time by group interaction, *F*[1, 48] = 10.13, *p* = .003, η^2^ = .17. Follow-up tests indicated that the Training group showed a significant improvement in the recognition of anger after training, *F*[1, 48] = 10.16, *p* = .003, η^2^ = .18; the Control group did not differ between pre- and posttest, *F*[1, 48] = 1.63, *p* = .21, η^2^ = .03. At posttest the Training group recognized significantly more angry expressions than the Control group, *F*[1,48] = 19.22, *p* < .001, η^2^ = .286, although there was no difference at pretest, *F*[1, 48] = .609, *p* = .439, η^2^ = .013.

#### Happiness

There was a significant main effect of group, *F*[1, 48] = 4.99, *p* = .030, η^2^ = .094, with the Training group recognising more happy faces. However, there was no effect of time, F[1, 48] = 0.75, p = .391, η^2^ = .01, and no interaction between time and group, F[1, 48] = 1.87, p = .178, η^2^ = .04. This shows that the groups did not improve significantly over time.

#### Disgust

There was no main effect of time, *F*[1,41] = 0.88, *p* = .35, η^2^ = .02, or group, *F*[1,41] = 0.00, *p* = .97, η^2^ = .00, and no significant interaction between time and group, *F*[1,41] = 0.39, *p* = .54, η^2^ = .01.

### Reoffending data 6 months after testing

**Fig 2:**
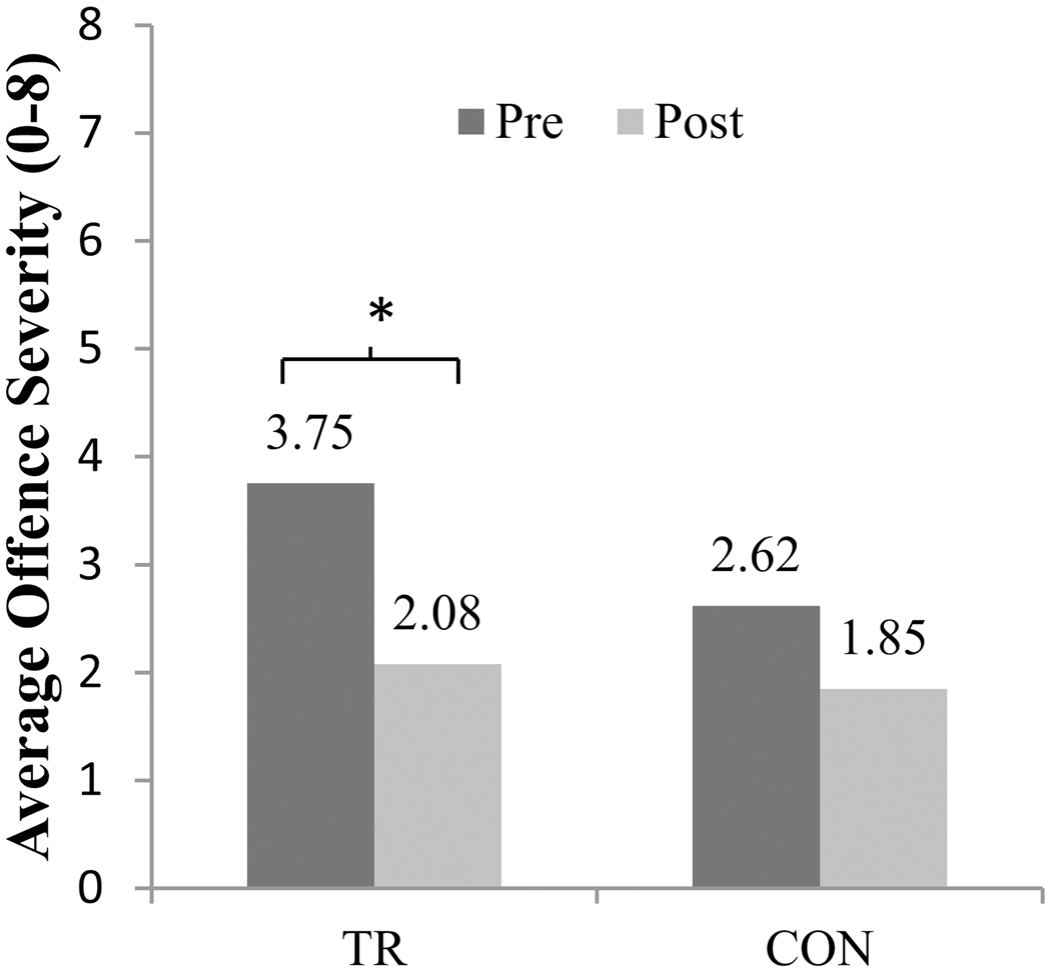
Effect of emotion training on crime: Offence severity 6 months before (pre) and 6 months after (post) for young offenders in the Training (TR) or Control (CON) group.

Posttest reoffending data are shown in Table 1. In the 6 months after the training 12 offenders in the Training group and 10 offenders in the Control group reoffended. There were no differences between the groups in offence frequency (z = 1.02, *p* = .31) or severity, *F*[1, 49] = 0.12, *p* = .73. A random effects negative binomial model revealed a significant reduction in offence rates from the pre- to post-training 6-month periods for both groups (z = -3.45, *p* < .01). However, paired samples t-Tests and general linear models adjusting for baseline differences from the mean only showed significant reductions in reoffending severity for the Training group (re-offence mean severity: *t*[23] = 2.17, p = .04; re-offence most severe: *t*[23] = 2.82, p = .01; B = -0.35, z = -2.07, *p* = .04; see Fig 2). There was no difference between groups in the time it took to reoffend (*z* = 0.70, *p* = .48), accounting for right side censoring and time in custody. Eleven offenders were in custody in the 12 months of the assessment period, three in the six months period before (2 Training, 1 Control) and eight in the 6 months follow-up period (4 Training, 4 Control). These subgroups did not differ in the number of days spent in custody, and nor was being in custody associated with the number or severity of crimes committed (*p*s > .05).

## Discussion

The present study sought to establish whether emotion recognition abilities could be improved in young offenders, a group that has been found to have particular problems in recognising negative emotions [19]. An intervention that has been shown to improve emotion recognition in individuals with acquired brain injury [28] was used and emotion recognition performance of offenders receiving emotion training was compared to a matched control group of offenders not receiving training. This study is the first to investigate whether emotion training can positively affect subsequent reoffending as assessed through objectively recorded crime data.

The findings show that juvenile offenders in the Training and Control groups displayed statistically equivalent recognition abilities at pretest and confirm previous findings of poor recognition of negative emotions in offenders. Bowen et al. [19] compared young offenders (YO) to matched controls (MC) and reported the following recognition rates: Fear: YO = 35%, MC = 39%; Sadness: YO = 46%, MC = 52% Anger: YO = 50%; MC = 55%. Happiness: YO = 81%; MC = 81%. We also demonstrated that the emotion training had a significant positive effect on emotion recognition scores in the subgroup of offenders that received the training. There was a significant improvement in the recognition of fear and sadness, two emotions known to be difficult to recognise in antisocial individuals, as well as in anger, with recent evidence suggesting that young offenders may particularly struggle to detect lower intensity angry faces [19]. Importantly, these improvements were specific to the Training group and not due to repeated testing, because offenders in the Control group showed either no improvement (fear, anger) or got worse (sadness) in recognising these emotions. It is not surprising that there was no significant improvement in happiness recognition since young offenders generally do not show impairments in detecting this emotion, also confirming previous results [15, 25]

Previous studies have found disgust recognition impairments in antisocial children [16] and our results did show impaired disgust recognition at pretest. However, disgust did not form part of the training and no improvement was observed over time. Our results therefore indicate that improvement in emotion recognition was specific to those emotions that were trained. This finding is in contrast to Schonenberg et al. [29], who found that the implicit training of violent offenders to attend to the eye areas of fearful faces, which gradually decreased in intensity, led to a better detection of fear but also improved sensitivity to other basic emotions. We suggest a number of subtle differences in the design of the two studies, which may account for these differing results. Firstly, we measured facial expression recognition using still images of varying intensities and recorded percentages of correct responses, whereas Schonenberg et al. measured emotion recognition using dynamic emotion expressions. Secondly, our sample included young offenders in general, whereas Schonenberg et al. utilised a sample of violent offenders high in psychopathic traits whose impairments may be qualitatively different. Finally, Schonenberg et al. found that only an implicit task, which gradually decreased in intensity led to improvements whereas training to increase attention to the eye without decreases in intensity did not improve facial recognition. This highlights important differences in training designs. Since our training was explicit in teaching young offenders how to identify key facial features belonging to certain emotional expressions, and not just to (only) pay attention to the eye regions, it is may be not surprising that our training effects did not spillover to different, untaught emotions. We believe this difference could be qualitatively important and may result in more prolonged learning and therefore have a greater impact on behaviour than any method that only focuses on implicit learning. Nonetheless future studies comparing improvements associated with different training techniques would be beneficial to clarify these differences.

Turning to the crime data, we found that offenders in the Training and Control groups were similar with respect to lifetime crime data (as well as age, IQ, SES and CU traits) before the study, but differed in offence severity in the 6 months before the training, with those who went on to take part in the emotion training having committed more severe offences (*d* = 0.55). When we examined crime data in the 6 months following the training, we found that both groups exhibited significant reductions in reoffending rates. Given that all juvenile offenders continued to be closely monitored and to receive their ‘treatment as usual’ by the YOS during this period, it is unsurprising that both groups of offenders showed a reduction in offending rates. However, we found that only young offenders who participated in the emotion training showed a significant reduction in the severity of the crimes they committed. Taking Blair’s [10] theory into account, it is not surprising that the improvements relate specifically to offence severity. High severity crimes generally involve more physically aggressive behaviour and more interpersonal violence compared to less severe crimes that typically include theft and criminal damage. It is possible that the improvements in the recognition of angry, sadness and fear in others as a result of the training resulted in a better understanding of the emotions of potential victims and thus a reduction in physical aggression and the commitment of severe offences.

### Limitations

It should be noted that the observed effects, in terms of offences severity, were relatively small and of moderate effect size. This is understandable in a sample as small and complex as the current one. Most of the young offenders were persistent offenders (see Table 1 for lifetime crime data) and therefore by definition those whose offending trajectory is resistant to change. In addition, the training time was relatively short. We consider this to be a major benefit of the program, demonstrating how a short, quick and easy to administer intervention can have positive results. Nevertheless, a previous study using the same intervention in a sample of patients with severe traumatic brain injury [28] allocated significantly more time to complete the training (9 hours). The nature of that sample may be responsible for the longer duration. However, it may also be that in order to obtain larger effects, a longer and more advanced program could be beneficial in young offenders. This could include the training of additional emotions such as disgust, that have been shown to be related to antisocial behaviour, and the training of how to respond to others who are afraid, sad, and angry.

The intervention was not randomised. Because of logistical issues within the YOS it was not possible to randomly allocate offenders to group; consequently, a quasi-experimental design was used based on the availability of young offenders to attend the YOS offices. With the help of caseworkers it was decided in advance whether offenders would be able to attend for the number of sessions required to complete training. Those who were unlikely to be able to attend all sessions formed the control group. It could be argued that this could have biased the sample; however, we have shown that the two groups did not differ on a number of key variables suggesting the groups were equal. It should be noted that there are other possible confounding variables, which we did not control for and that could have impacted upon our data, such as substance use, self-reported aggression, opportunity, and maltreatment. Our findings should therefore be interpreted with this caveat in mind. Despite the drawback of non-randomised designs, evidence suggests that there are no differences in magnitude of effect sizes between randomised control trials and quasi-experimental designs [36]. Furthermore, according to The Maryland Scientific Methods Scale [37], the current intervention reflects a Level 3 intervention program to the extent that we measured of crime before and after the training programme, using a comparable control group, whilst controlling for other variables that influence crime. A supportive Level 3 evaluation provides evidence that an intervention is promising. Future research will need to confirm these results within a randomised control trial framework. Finally, our study does not explain why the emotion training improved expression recognition and reduced reoffending severity. Future research should employ techniques like eye-tracking methodologies to establish why and how improvement is achieved. This may also help to clarify whether the recognition impairments in offenders are caused by abnormal attention or scanning patterns.

### Clinical Implications

We have shown that emotion recognition can be improved in youths who come into contact with the police for a wide range of different types of antisocial behaviour problems by administering a relatively brief, targeted intervention. Importantly, the training subsequently had a positive effect on criminal behaviour by reducing reoffence severity. This suggests that interventions that target neurobiological impairments are not only relatively easily achievable, but also have a beneficial impact on the lives of young people and their communities.

Within the autism literature compensatory changes in neural activity, measured by fMRI, have been observed alongside improved recognition in those with autism trained to attend and interpret emotional faces [38]. It is therefore possible that targeted trainings such as the one implemented here affect the neural processes involved in emotion recognition and thereby achieve long-term behavioural change, particularly in a young sample in which the brain is still developing [39]. If this training can alter young offenders’ neural activity and produce improvement in recognition, it would provide a cost-effective and relatively quick way of managing a population of individuals whose combined offending produces the majority of harm in their communities.

## Supporting Information

S1 Dataset SPSS.xls file containing dataset underlying reported findings. (XLS)
